# Diagnosis and Treatment of Paracoccidioidomycosis in the Maxillofacial Region: A Report of 5 Cases

**DOI:** 10.1155/2018/1524150

**Published:** 2018-04-18

**Authors:** Antonio Dionízio De Albuquerque Neto, André Vitor Alves Araújo, Daniel Assunção Cerqueira, Lorenzzo De Angeli Cesconetto, Nilton Provenzano, Eder Magno Ferreira de Oliveira

**Affiliations:** Oral and Maxillofacial Surgery, Dr. Mário Gatti Municipal Hospital, Campinas, SP, Brazil

## Abstract

Paracoccidioidomycosis is a fungal infection initially described in Brazil in 1908 by Adolfo Lutz. Manifestation of this disease depends on numerous factors, such as microorganism virulence, hormonal profile, genetic predisposition, nutrition, and immune system. It is characterized classically by cutaneous lesions, lymphadenopathy, and pulmonary involvement. It presents male predilection, which can be explained by the protective effect of female hormones. Classic presentation within oral cavity is a superficial ulcer characterized by hemorrhagic dots of moriform aspect. Treatment should be established individually and closely monitored. This manuscript aims at reporting five cases of this pathology.

## 1. Introduction

Paracoccidioidomycosis (PMC) is a fungal infection caused by *Paracoccidioides brasiliensis*, and it affects mainly patients living in the South and Central America. It has male predilection (M : F ratio was 15 : 1), preferably middle-aged rural workers (30 to 50 years) [1, 2].

Primarily, PMC occurs in the lung due to the mechanism of aspiration of spores, which can be spread through lymphatic or hematogenous routes to other organs of the body. Oral lesions manifest as ulcers with hemorrhagic dots of moriform aspect. Clinically, due to healing difficulty, differential diagnoses of PMC are squamous cell carcinoma, traumatic ulcer, lymphoma, oral tuberculosis, sarcoidosis, Wegener's granulomatosis, leishmaniasis, actinomycosis, and primary syphilis [1, 3–7].

Treatment is based on three groups of drugs: amphotericin B (from the group of polyenic antibiotics); sulfadiazine and other sulfanilamide compounds; and the group of azolic drugs. Duration of treatment varies from 6 to 24 months, and if not treated properly it can be fatal [5, 8, 9].

This study aims at reporting five cases of paracoccidioidomycosis in the facial region, diagnosed and treated in a hospital in the countryside of São Paulo, Brazil. The first case will be exposed in details, and other cases will be summarized on Table 1.

## 2. Case 1

A 47-year-old male patient who was a rural worker reported “wounds in the mouth” and persistent cough for about 6 months. He reported chronic smoking and denied comorbidities.

**Figure 1:**
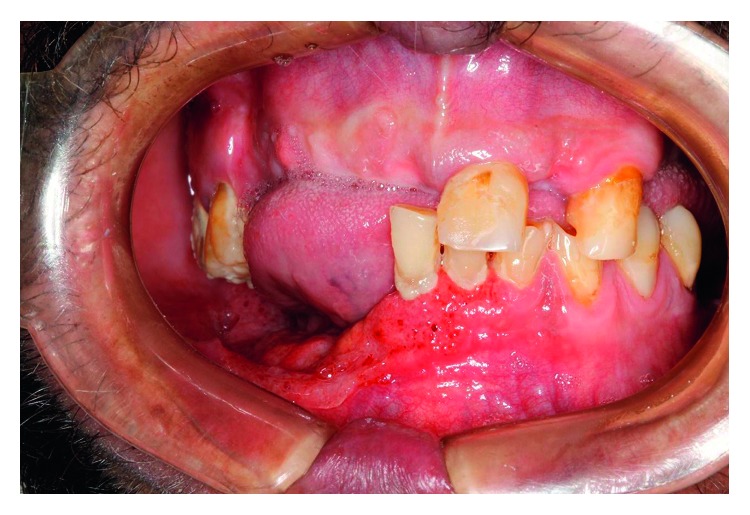
Initial intraoral clinical appearance showing the moriform aspect in the right buccal floor and alveolar ridge.

**Figure 2:**
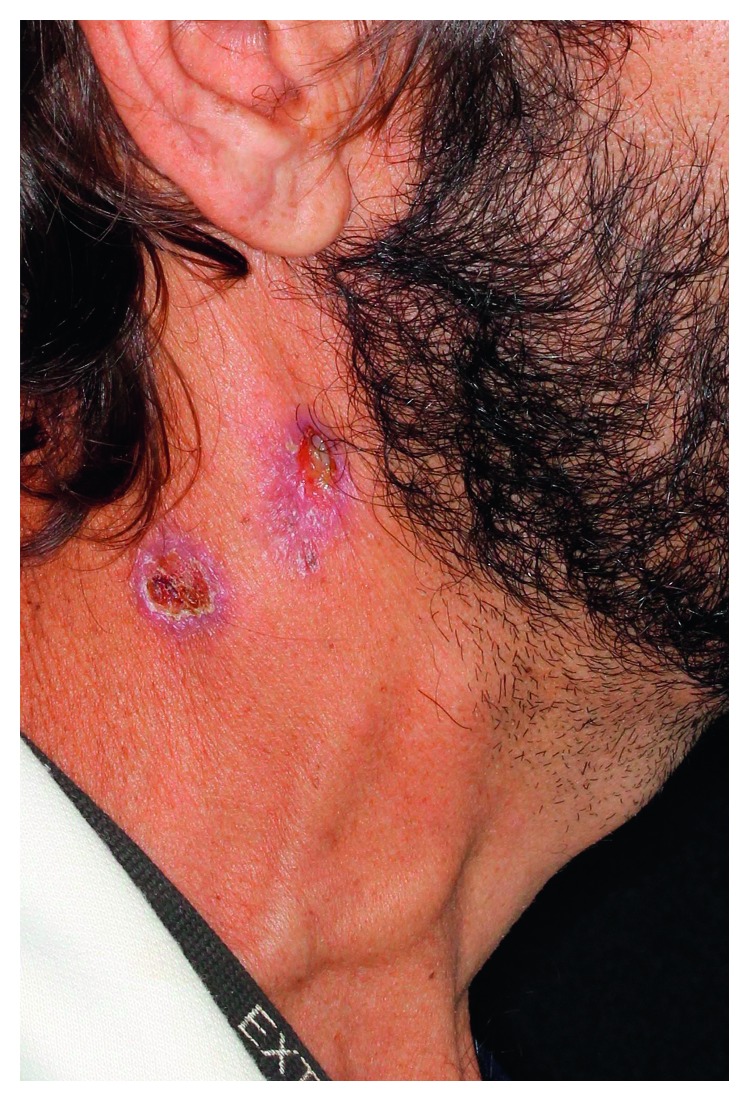
Ulcerated lesion in the right cervical/submandibular region.

**Figure 3:**
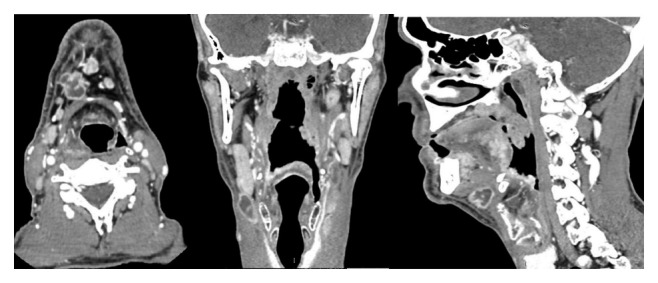
Computed tomography cuts showing lymph node enlargement as a hypodense and heterogeneous image in the right submandibular region.

A granulomatous ulcer with hemorrhagic dots and moriform aspect was observed in the posterior mandibular alveolar ridge and in the right jugal mucosa (Figure 1). Some dental elements presented severe mobility, which began after lesion emergence. He had an ulcerated lesion in the right cervical region with serous exudate (Figure 2). Cervical contrast CT scan revealed images compatible with cervical lymphadenopathy in the right side and exteriorization of the infectious process through cutaneous fistula (Figure 3). Chest X-ray showed infiltrative lesions with reticulonodular aspect in butterfly-wing shape.

A direct mycological examination was performed from cervical lymph node puncture, sputum, and intraoral mucosal scraping. The result was positive for the presence of yeasts with multiple buds surrounding mother cells, suggestive of *P. brasiliensis*. Thus, culture with fungal isolation was carried out, and the final result was *Paracoccidioides* spp. DNA-PCR examination for *Mycobacterium tuberculosis* was negative, and the result was nonreagent for the rapid HIV test, revealing leukocytosis (13,500/mm³), without left deviation.

**Figure 4:**
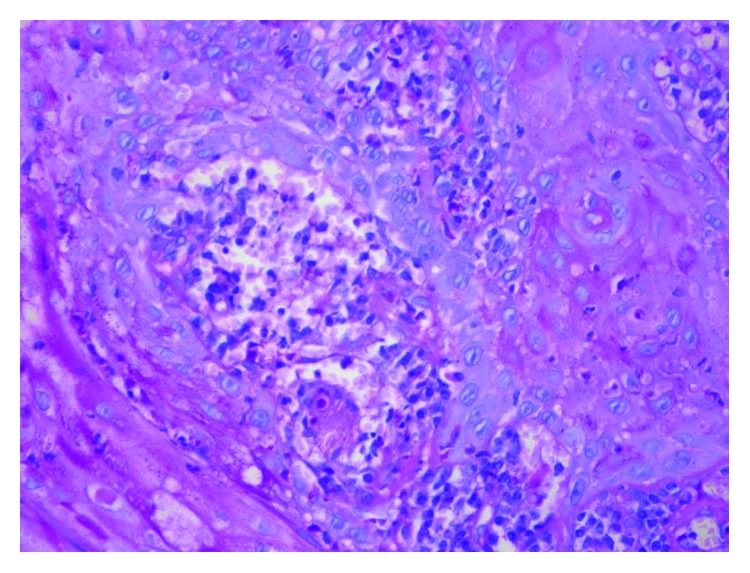
Anatomopathological examination image.

Incisional biopsy of the intraoral lesion was performed under local anesthesia. Histological sections revealed a mucosal fragment coated with parakeratinized stratified squamous epithelial tissue, exhibiting pseudoepitheliomatous hyperplasia constituted by dense connective tissue. An intense granulomatous inflammatory infiltrate with predominance of eosinophils and formation of granulomas and microabscesses were observed, as well as numerous multinucleated giant cells. Spherical fungi of different diameters and birefringent membrane located both within multinucleated giant cells or dispersed through the tissue were also present. In depth, there were fragments of mineralized tissue and hemorrhagic areas (Figure 4).

**Figure 5:**
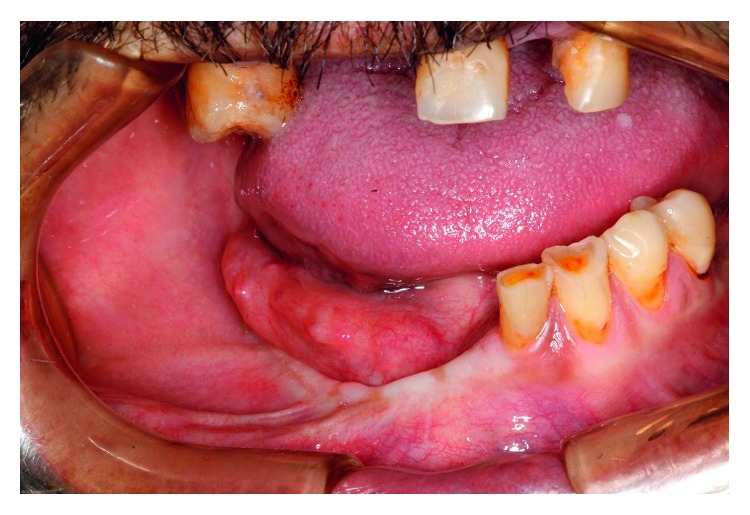
Intraoral oral appearance after antifungal therapy demonstrating lesion regression.

**Figure 6:**
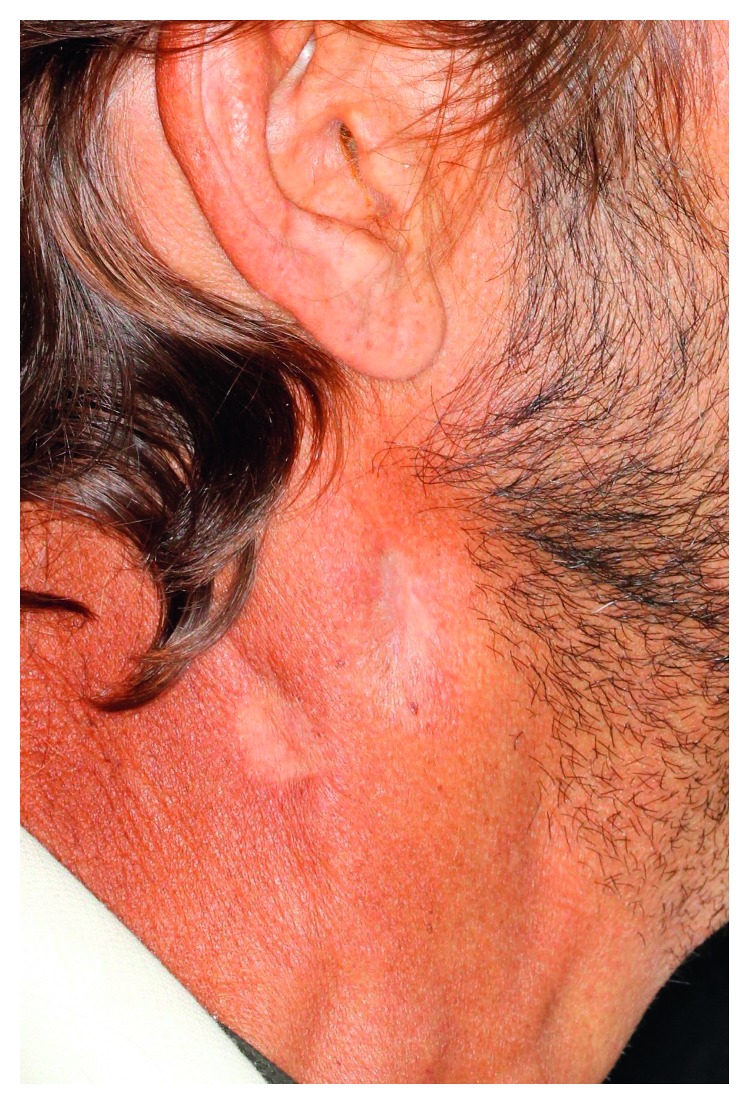
Clinical aspect of the submandibular/cervical region showing the remission of the ulcerated lesion and associated fistula.

The patient was referred to an infectologist who followed up the case. An association of sulfamethoxazole 2400 mg + trimethoprim 480 mg was prescribed three times a day during 12 months. After 1-year follow-up, the patient was asymptomatic and presented remission of the oral and cervical lesions (Figures 5 and 6).

## 3. Case 2

**Figure 7:**
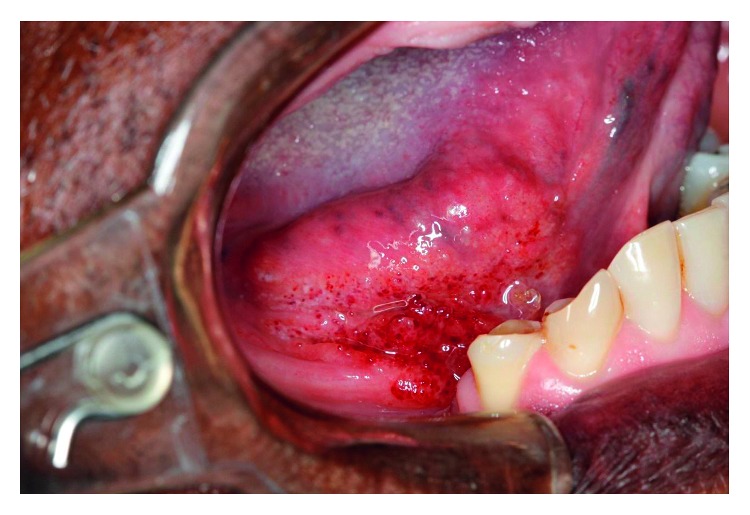
Classical presentation of PMC as exuberant hemorrhagic dots.

A 68-year-old male patient who had melanoderma and was a chronic smoker and resident of the rural region was referred to the surgery outpatient clinic with “mouth sore” complaint. Clinical examination verified an ulcerated lesion with hemorrhagic dots, a classic aspect of PCM (Figure 7). Biopsy was performed under local anesthesia, and the hypothesis was confirmed. Itraconazole therapy was initiated by the infectious team.

## 4. Case 3

**Figure 8:**
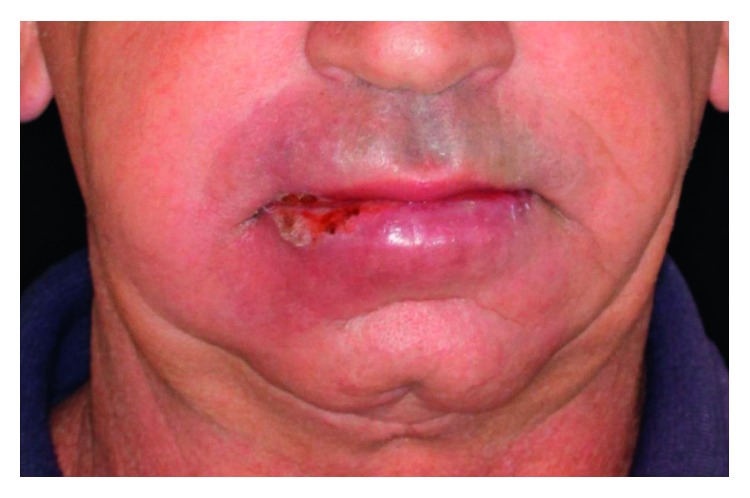
Frontal view of the patient presenting ulcers on the lips and associated cutaneous erythema.

**Figure 9:**
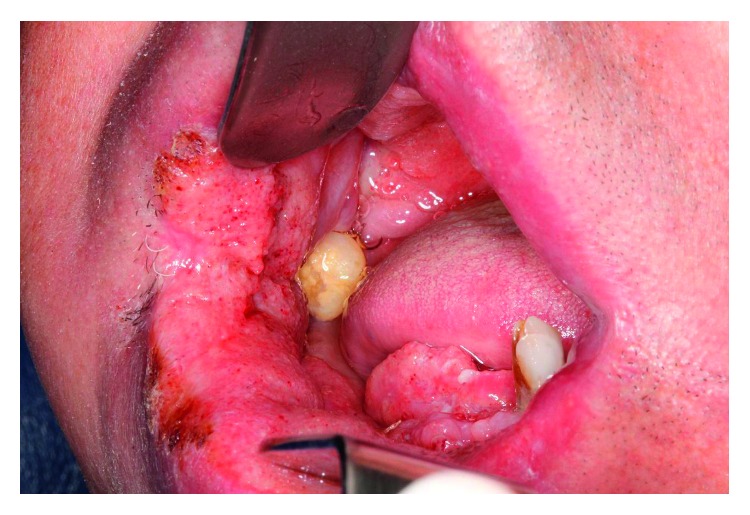
Ulcerated lesion in the jugal mucosa, lips, and buccal commissure.

A 57-year-old male patient who was a rural worker and smoker with systemic arterial hypertension attended the outpatient clinic complaining about “sore and pain in the lips and mouth.” Clinical examination revealed the presence of erythematous ulcers in the lips and oral mucosa; the patient had a slight limitation of mouth opening due to the inflammatory process installed in the lips (Figures 8 and 9). Diagnostic suspicion of malignant neoplasia or paracoccidioidomycosis was raised. After anatomopathological examination of multiple fragments collected under local anesthesia, PMC was confirmed. Antifungal therapy with Bactrim (trimethoprim + sulfamethoxazole) was conducted.

## 5. Case 4

**Figure 10:**
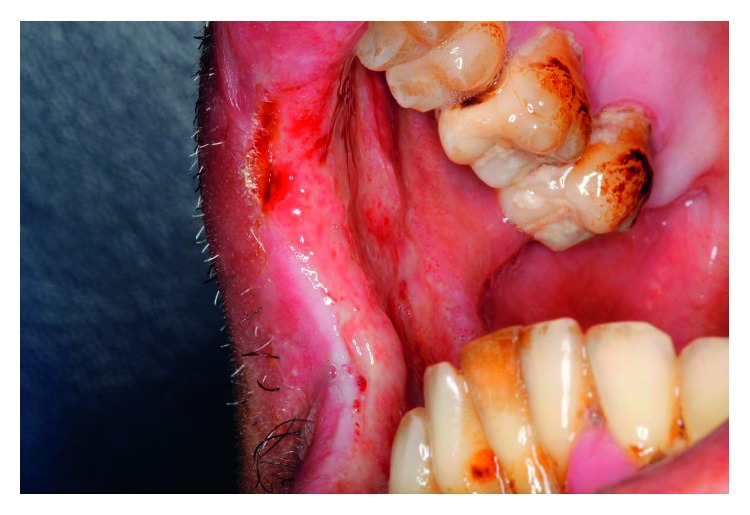
Ulcerated lesion of approximately 4 cm in the right jugal mucosa and buccal commissure.

A 48-year-old male patient who was a rural worker presented to the clinic, and he denied habits, addictions, or comorbidities. His main complaint was a “bleeding wound in the mouth.” Clinical examination revealed an ulcer lesion of approximately 4 cm associated with hemorrhagic dots (Figure 10).

PMC was confirmed after anatomopathological examination. Therapy started promptly with trimethoprim + sulfamethoxazole.

## 6. Case 5

**Figure 11:**
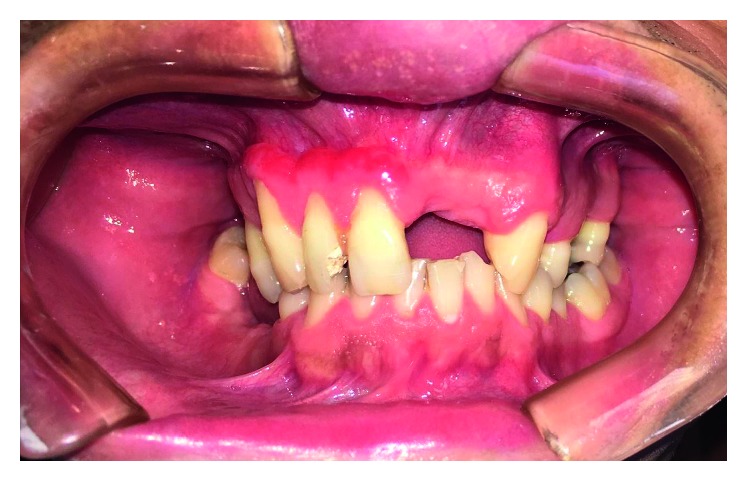
Erythematous lesion in the gums of the right maxilla and associated dental mobility.

A 38-year-old male patient who was a rural worker and smoker presented to the clinic, and he denied comorbidities and complained about gingival bleeding when he brushed his teeth (Figure 11). Biopsy was performed since the lesion did not disappear after basic periodontal treatment. Pathologic examination revealed PMC; thus, antifungal therapy with Bactrim was initiated.

## 7. Discussion

Paracoccidioidomycosis was first described in Brazil in 1908 by Adolfo Lutz. Years later, in 1911, Alfonse Splendore reported other clinical cases and studied in detail its microbiology. PMC is also known as Brazilian blastomycosis, South American blastomycosis, Lutz's disease, Lutz–Splendore–Almeida disease, and Lutz's mycosis. Despite being a disease that does not require compulsory notification in Brazil (absence of precise data of its incidence), most cases were reported in the southern, southeastern, and center-western regions, closely related to rural dwellers. All patients listed in Table 1 reside in the countryside [7, 8].

Infective propagules (microconidia) reach the lower airways, where they constitute a primary complex, and possible dissemination of the fungus via lymphatic vessels and hematogenous spread to other organs are likely to happen. Manifestation of the disease depends on numerous factors, such as microorganism virulence and hormonal, genetic, nutritional, and immune system issues. Approximately 10 million people are infected worldwide by this fungus, but only 2% develop the active disease. Although transmission between humans is possible, it is very rare because the fungi switch to yeast form which cannot pass through the cell wall, differently from the unmodified mycelial form found in the nature [4, 5, 10, 11].

PMC has predilection for male gender in a ratio of 15 : 1; this difference can be explained by the protective effect of female hormones. Beta-estradiol seems to have a protective effect which inhibits transformation of the mycelium into yeast. Among individuals under 14-year-old or during menopause, the microorganism does not present predilection to gender due to decreased hormonal influence. Bicalho et al. [12] observed in a sample of 62 Brazilian patients the ratio of 30 : 1 between men and women. All five cases reported in this article were from male patients [1, 8, 13, 14].

The classic presentation of PMC in the oral cavity is a superficial ulcer characterized by hemorrhagic dots that usually affect the gums, palate, and mucous membranes. When ocular and genital mucosae are affected, they present the same characteristics of the classic oral lesion. Gingiva may be erythematous with consequent periodontal bone loss, mobility, and tooth loss. Bone destruction can be perceived through panoramic radiography, usually characterized by osteolytic areas in the alveolar region. This bone involvement can reach the maxillary sinus causing its opacification and destruction of the anterior wall. Palate perforation with oral-nasal communication rarely occurs. Cutaneous lesions can be caused by continuity, hematogenous contamination or, less often, by direct inoculation; in addition, contamination might happen through fistulas related to infected lymph nodes or osteomyelitis. Lesions in the nasal region are described in the literature; usually, they are continuity of lesions in the upper lip, mainly as a consequence of macrocheilia or related to direct contamination by sinus drainage [1–6, 15].

Lymph nodes are more frequently involved in young patients (less than 30 years old); they are initially firm, painful, and hypertrophic, and later, they develop phlogistic signs and suppuration [1, 2]. In case 1, the patient evolved with cutaneous lesion associated with cervical fistula in the right side and lymph node involvement, and the infection by ganglionic paracoccidioidomycosis was confirmed by aspiration. Cervical chain involvement was evident in the CT with contrast.

Bones and joints can be affected in about 6–20% of the reported cases, which is usually asymptomatic. Bone/joint lesions are predominant in the thorax, scapular region, and upper limbs. Paracoccidioidomycosis can affect any organ/region, including the central nervous system, for example, as cerebral granuloma and meningoencephalitis. Thus, possible lesions should be investigated through imaging and clinical findings [2, 5, 16]. In our article, three cases (60%) evolved with alveolar osteolysis, substantial dental mobility, and multiple dental losses.

Reports of paracoccidioidomycosis in HIV patients are less frequent than expected; this is still not well understood, but epidemiological differences between HIV and *P. brasiliensis* infections among rural and urban populations may be related. Morejón et al. [17] observed a discreet association between HIV and *P. brasiliensis* in only 1.4% of the analyzed cases. In general, the patients were farmers and the majority had less than 200 CD4+/*μ*L. Thus, PMC can be considered an opportunistic infection among HIV positive population of endemic regions. Eight cases were reported among immunosuppressed patients after renal transplantation, in which latent foci were reactivated due to immunologic depression. There was no association with immunosuppression in the cases reported in our article [2, 13].

Radiographic findings in the posteroanterior thoracic incidence consist of reticulonodular pulmonary infiltrate, mainly in the upper two-thirds; it was bilateral and asymmetric. Computed tomography of the thorax shows nodules, ground-glass opacities, acinar lesions, parenchymal bands, peribroncovascular interstitial thickening, paracicatricial emphysema, and traction bronchiectasis [8, 16]. Similar findings were found in two patients in our study of cases (40%).

Facing the infection by paracoccidioidomycosis, an immune response is generated as granulomatous epithelial growth, which is a way to contain the progression of the fungal infection. This structure is formed by macrophages that surround the pathogen. Anatomopathological examination revealed an ulcer with pseudoepitheliomatous hyperplasia, as well as a granulomatous structure composed by giant multinucleated cells and macrophages. Fungi can be found inside giant cells or free. This fungus classical appearance is similar to “Mickey Mouse ears” or “boat steering wheel,” which represents multiple yeasts with associated buds [1, 4, 5].

Diagnosis can be achieved through some complementary exams, such as direct microscopy and mycological examinations, which aim to identify microorganism in the sputum, purulent exudate, or material collected from lesions. Fungal culture may be useful but impaired due to slow fungal growth or sample contamination and its low sensitivity. Visualization of fungal particles can be obtained from pathological examination with Gomori–Grocott dye or PAS (periodic acid-Schiff). Exfoliative cytology is a diagnostic option, and it is also interesting to follow-up infection evolution during treatment. More recent options aim to identify antigenic/antibodies material and to evaluate quantity/type of antibodies present, such as direct/indirect immunofluorescence, immunoelectrophoresis of serum proteins, immunodiffusion technique, ELISA, and immunoblotting/western blot. In general, PCR to identify *P. brasiliensis* DNA has high sensitivity and specificity [1, 4, 5, 7].

Treatment should be instituted on an individualized basis and rigorous follow-up; special attention should be given to comorbidities, nutritional status, and drug use by patients starting treatment, besides, adherence of the patient to the pharmacotherapy. Medications of choice depend on the severity of the condition. Itraconazole is considered the gold standard in mild and moderate conditions, with a dosage of 200 mg/day for 6 to 18 months. Combination of sulfamethoxazole 2400 mg + trimethoprim 480 mg (Bactrim), daily, from 12 to 24 months is a viable option with excellent results. In severe cases, amphotericin B is the drug of choice, at doses of 0.75 mg/kg/day; it presents high toxicity, side effects, possible hospitalization, and renal function monitoring. *In vitro* studies demonstrate strains of *P. brasiliensis* resistant to various types of drugs; thus, new treatment protocols are necessary [2, 4–7, 9, 12, 15]. In our case reports, 80% of patients were treated successfully with the combination of sulfamethoxazole + trimethoprim.

Disease cure is difficult due to the risk of relapse; treatment success must be analyzed through clinical, radiographic, and immunological examination. Recurrence should be considered, although it is more common among immunosuppressed patients. Estimated mortality rate is 30% among patients with HIV, which can be decreased with early treatment with amphotericin B followed by life-long prophylaxis with some antifungals from the group of azoles or sulfonamide. Vaccine based on *P. brasiliensis* GP43 antigen requires more studies and greater applicability [4, 5, 13, 14].

## 8. Final Considerations

Paracoccidioidomycosis represents an important public health problem due to its high morbidity and mortality potential. Long and costly treatments in addition to recurrent episodes demand PMC treatment innovations. It should be considered as differential diagnosis in cases of opportunistic infection in immunosuppressed patients. Our study reported a range of presentations of the same infection, from complicated cases to cases that did not present classically as PMC.

## Figures and Tables

**Table 1:** Details of the clinical cases reported in the study.

Case	Gender	Age	Habitation	Habits andaddictions	Treatment	Comorbities	Anatomic region	Follow-up
1	Male	47	Rural	Drug addiction,alcoholism,and smoking	Sulfamethoxazole + trimethoprim	Malnutrition	Oral mucosa + pulmonary + lymph node	18 months
2	Male	68	Rural	Smoking	Itraconazole	Negative	Oral floor	12 months
3	Male	57	Rural	Smoking	Sulfamethoxazole + trimethoprim	Arterial hypertension	Lips + oral mucosa	24 months
4	Male	48	Rural	Smoking	Sulfamethoxazole + trimethoprim	Negative	Oral mucosa	20 months
5	Male	38	Rural	Smoking	Sulfamethoxazole + trimethoprim	Negative	Alveolar ridge	8 months

## References

[1] Neville B. W., Damm D. D., Allen Jeb C. M. (2016). *Oral and Maxillofacial Pathology*.

[2] Marques S. A., Leite R. C., Paulo S. (2012). Paracoccidioidomycosis. *Clinics in Dermatology*.

[3] Regezi J., Sciubba J. (2012). *Patologia Oral Correlações Clinicopatológicas*.

[4] Ameen M., Talhari C., Talhari S. (2009). Advances in paracoccidioidomycosis. *Clinical and Experimental Dermatology*.

[5] Abreu M. A. D., Salum F. G., Figueiredo M. A., Cherubini K. (2013). Important aspects of oral paracoccidioidomycosis–a literature review. *Mycoses*.

[6] Girardi F. M., Scrofernecker M. L. (2015). Clinical image oral paracoccidiodomycosis mimicking lip carcinoma. *Brazilian Journal of Infectious Diseases*.

[7] Palmeiro M., Cherubini K., Yurgel L. S. (2005). *Paracoccidioidomicose–Revisão da Literatura*.

[8] Wanke B., Aidê M. A. (2009). Paracoccidioidomycosis. *Jornal Brasileiro de Pneumologia*.

[9] Shikanai-Yasuda M. A. (2015). Paracoccidioidomycosis treatment. *Revista do Instituto de Medicina Tropical de São Paulo*.

[10] Talhari C., Souza J. V. B. D., Parreira V. J., Reinel D., Talhari S. (2007). Oral exfoliative cytology as a rapid diagnostic tool for paracoccidioidomycosis. *Mycoses*.

[11] Azenha M. R., Caliento R., Brentegani L. G., Lacerda S. A. D. (2012). A retrospective study of oral manifestations in patients with paracoccidioidomycosis. *Brazilian Dental Journal*.

[12] Bicalho R. N., Santo M. F., Aguiar M. C. F., Santos V. R. (2001). Oral paracoccidioidomycosis: a retrospective study of 62 patients. *Oral Diseases*.

[13] Radisic M. V., Linares L., Afeltra J. (2017). Acute pulmonary involvement by paracoccidiodomycosis disease immediately after kidney transplantation: case report and literature review. *Transplant Infectious Disease*.

[14] De Azevedo Izidoro A. C. S., Silva P. C. D., Ribas M. D. O. (2007). Case of recurrent paracoccidioidomycosis in female 10 years after initial treatment. *Bulletin of Tokyo Dental College*.

[15] Neto S. S., de Paulo L. F. B., Rosa R. R. (2012). Oral paracoccidioidomycosis as a differential diagnosis of oral cancer. *Images in Infectious Diseases*.

[16] Lima Júnior F. V. A., Savarese L. G., Monsignore L. M., Martinez R., Nogueira-barbosa M. H. (2015). Computed tomography findings of paracoccidioidomycosis in musculoskeletal system. *Radiologia Brasileira*.

[17] Morejón K. M. L., Machado A. A., Martinez R. (2009). Paracoccidioidomycosis in patients infected with and not infected with human immunodeficiency virus: a case-control study. *American Society of Tropical Medicine and Hygiene*.

